# Isolation, genomic characterization, and pathogenicity of an emerging PEDV variant in Korea related to virulent Chinese strains

**DOI:** 10.3389/fvets.2026.1785848

**Published:** 2026-04-01

**Authors:** Dong-Kyu Lee, Jonghyun Park, Seung-Chai Kim, Hwan-Ju Kim, Jung-Hoon Kwon, Young S. Lyoo, Won-Il Kim, Choi-Kyu Park, Hye-Ryung Kim

**Affiliations:** 1 Institute for Veterinary Biomedical Science, College of Veterinary Medicine, Kyungpook National University, Daegu, Republic of Korea; 2 Accumedix Inc., Iksan, Jeollabuk-do, Republic of Korea; 3 College of Veterinary Medicine, Jeonbuk National University, Iksan, Jeollabuk-do, Republic of Korea; 4 Department of Diagnostic Medicine/Pathobiology, College of Veterinary Medicine, Kansas State University, Manhattan, KS, United States

**Keywords:** isolation, pathogenicity, phylogenetic, porcine epidemic diarrhea virus, variant

## Abstract

**Introduction:**

The ongoing evolution of porcine epidemic diarrhea virus (PEDV) continues to significantly threaten the swine industry, necessitating extensive surveillance for the emergence of new PEDV variants in the field. Although genotype 2b (G2b) PEDV strains have been predominant in Korea since 2013, this study reports PEDV strains distinct from Korean G2b strains from two Korean pig farms experiencing severe diarrhea and mortality in sucking piglets.

**Methods:**

We report the first isolation and detailed characterization of a PEDV strain, designated KPED2021-1, from clinical specimens collected during outbreaks.

**Results:**

At the whole-genome level, KPED2021-1 demonstrated over 99G2c PEDV strains, which exhibit substantial genetic variation in China. In contrast, it shared 96.6%–98.7% homology with PEDV strains from Korea and other countries. Furthermore, KPED2021-1 showed greater nucleotide divergence in the NSP1, ORF3, and N genes from Korean and other global PEDV strains, while remaining more closely related to Chinese G2c strains in these regions. The amino-acid sequence of the spike (S) protein from KPED2021-1 was compared with that of the S protein from the Korean G2b strains, revealing 27 amino- acid substitutions and a unique deletion (11197). In experimental infection of suckling piglets, severe clinical symptoms and histopathological lesions were observed, resulting in a 100% mortality rate, suggesting that the KPED2021-1 strain is virulent.

**Discussion/Conclusion:**

This is the first report on the emergence of a G2c-subtype PEDV closely related to Chinese variants in Korea, and the isolate may serve as a useful reference strain for future vaccine development and cross-protection studies.

## 1 Introduction

Porcine epidemic diarrhea virus (PEDV), officially designated as *Alphacoronavirus porci*, is an enveloped, single-stranded RNA virus belonging to the *Alphacoronavirus* genus, within the *Coronaviridae* family. PEDV causes a highly contagious disease characterized by severe diarrhea, vomiting, and dehydration in pigs of all ages, and particularly high mortality in neonatal piglets (1, 2). Neonatal piglets are particularly susceptible due to their limited innate immune competence, slow enterocyte turnover, and complete dependence on maternally derived lactogenic immunity, especially secretory IgA in colostrum and milk (3). Insufficient induction or transfer of maternal mucosal antibodies markedly increases vulnerability to severe clinical outcomes, including dehydration-associated metabolic acidosis and high mortality. Since its first identification in Europe in the 1970s, PEDV has been responsible for recurrent epidemics and continues to cause major economic losses in the swine industry. Based on phylogenetic analyses of the spike (S) gene, PEDV strains are primarily divided into two genotypes: G1 (classical) and G2 (variant). Each genotype is further classified into subtypes, commonly G1a and G1b for G1, and G2a and G2b for G2 (2). Although classification criteria differ slightly among studies, sometimes producing inconsistencies in subtype names, the expanding database of submitted PEDV genomes, along with the continual reports of PEDV variants, have prompted proposals for additional subtypes (e.g., G1c and G2c) to capture genetically distinct clades (4, 5). In South Korea, highly virulent epidemic strains circulating since 2013 (e.g., KNU-141112) are classified as G2b under the domestic framework (6), whereas alternative classification systems designate these strains as G2c (5). Because the G2b nomenclature is widely used in the Korean epidemiological and vaccine context, this study follows the Korean classification system. Accordingly, predominant Korean epidemic strains are referred to as G2b, while genetically distinct Chinese-related variants are designated as G2c.

The PEDV S protein plays a crucial role in viral entry and pathogenicity, and mutations in this gene can significantly impact the virulence and tropism of the virus (7, 8). Previous studies have demonstrated that amino acid (aa) substitutions in the S protein, particularly nonsynonymous mutations in the S1 subunit and the fusion peptide region of the S2 subunit, can significantly alter the pathogenicity of PEDV, cell culture adaptation, and tropism, owing to these genetic variations in viral evolution and virulence (9–12). Therefore, mutations in the PEDV S protein should be continuously surveilled and characterized to anticipate the emergence of the variant strains and to understand their potential impact on viral pathogenicity and epidemiology.

PEDV was first reported in South Korea in the early 1990s, with initial outbreaks primarily classified as strains belonging to the classical G1 genotype (13). For nearly two decades thereafter, PEDV infections occurred sporadically and were effectively controlled through the use of attenuated vaccines derived from G1 strains. However, between 2013 and 2014, South Korea experienced a drastic surge in severe PED outbreaks coinciding with the introduction of highly virulent variant strains classified within the G2 genotype. These G2 strains are closely related to those responsible for major PED epidemics in China and the United States in 2010 and 2013, respectively, marking a genotype shift that has profoundly impacted PED epidemiology in Korea (14). Since the emergence of G2 PEDV, recurrent outbreaks have been continuously documented in South Korea, with notable epidemic waves reported during the winter seasons of 2013–2014, 2017–2018, 2018–2019, and 2021–2022, as recorded by the Korean Animal Health Integrated System (KAHIS). In addition, recent molecular epidemiological studies that analyzed the genetic characteristics of field-derived G2 strains in South Korea revealed that these viruses share close phylogenetic relationships with previously reported domestic strains, maintaining similar genotypic features despite some characteristic mutations (6). To date, highly pathogenic PEDV strains genetically or phylogenetically distinct from these domestic G2b strains have not been reported in South Korea.

In contrast, since 2010, China has experienced the continuous emergence and diversification of PEDV variants. Following the initial spread of a highly virulent G2 strain in central China, commercial vaccines based on the classical CV777 (G1), ZJ08 (G1), and AJ1102 (G2) strains had been widely used to more effectively control the disease (15, 16). Nonetheless, recent studies have reported the emergence of G2 variants capable of breaking through existing vaccine-induced immunity, often accompanied by severe disease and high mortality in neonatal piglets (17, 18).

Recent studies in China indicate that PEDV evolution involves not only point mutations but also recombination among co-circulating strains. Recombination within the S gene and other genomic regions has been documented in field isolates, contributing to the emergence of genetically mosaic variants with altered pathogenic or antigenic characteristics (4, 5, 17). In endemic settings where multiple lineages co-circulate, mixed infections may facilitate genetic exchange and accelerate viral diversification. Therefore, the introduction of a phylogenetically distinct PEDV lineage into a region where an endemic lineage is already established may alter the local evolutionary landscape and increase the complexity of genotype-based disease control strategies.

In this context, although several commercial PEDV vaccines are available for on-farm use in South Korea, PED outbreaks have persisted. Genetic analyses of PEDV isolates collected in South Korea between 2013 and 2022 have consistently shown that most strains circulating since 2014 belong to the G2b genotype. However, because farms are either unvaccinated or predominantly use vaccines based on classical G1 strains (19), it remains challenging to determine whether circulating G2b strains represent true vaccine-escape variants or whether continued outbreaks are primarily attributable to suboptimal matching between vaccine strains and field viruses. In such cases, PED outbreaks occurring in herds vaccinated with commercial G2b-based vaccines, despite the continued predominance of the endemic G2b lineage in Korea, necessitate comprehensive molecular characterization to clarify whether such cases reflect ongoing evolution within G2b or the introduction of genetically distinct PEDV lineages.

This study investigated PEDV strains associated with acute outbreaks that have occurred in two geographically distinct pig farms in Korea despite vaccination using commercial G2b-based vaccines in sow herds. Clinical samples obtained from affected piglets were subjected to molecular detection and whole-genome sequencing for viral characterization. The virus was subsequently isolated and designated KPED2021-1, followed by comprehensive genetic characterization using comparative genomic and phylogenetic analyses. In addition, experimental infection studies were conducted to evaluate the pathogenic properties of the isolate. Through this integrated approach, the present study aimed to clarify the genetic background and biological characteristics of the PEDV strain responsible for these outbreaks and to determine whether this strain represents a variant distinct from PEDV strains that previously circulated in South Korea. To the best of our knowledge, this study is the first to describe the isolation and provide a pathogenic characterization of a virulent PEDV strain in South Korea that does not belong to the endemic G2b.

## 2 Materials and methods

### 2.1 Clinical sample collection from affected farms

In October 2021 and March 2022, two cases of severe diarrhea due to suspected PEDV infections were identified in two different pig farms (Farms A and B) located in Chungcheongnam-do, in the central region of Korea. The two farms were located approximately 23.4 km apart and were epidemiologically unrelated. All sows on Farms A and B were routinely vaccinated using live and killed commercial G2b PEDV-based vaccines before the outbreak, following a vaccination regimen recommended in South Korea, which involves administering one oral prime dose of live attenuated vaccine, followed by two intramuscular booster doses of inactivated vaccine at 2–3 week intervals before farrowing. However, despite extensive vaccination against PEDV, typical clinical signs of acute PEDV infection were observed in both farms. Within a week of the onset of symptoms, almost all suckling piglets were culled, and approximately 80% of sows exhibited anorexia and diarrhea. The veterinarians in charge of both farms urgently submitted clinical samples to the diagnostic laboratory of Kyungpook National University for etiological diagnosis. Each sample was separately suspended in phosphate-buffered saline, homogenized, and centrifuged. Subsequently, the supernatant of each sample was used to extract total DNA/RNA using the Patho Gene-spin™ DNA/RNA Extraction Kit (iNtRON Biotechnology, Sungnam, Korea). The extracted nucleic acids were immediately used for cDNA synthesis, while the remainder was stored at −80 °C.

### 2.2 Molecular assays for screening enteric pathogens

To detect pathogens that cause or may cause diarrhea, real-time reverse-transcription polymerase chain reaction (qRT–PCR) was performed for nine viral pathogens, namely PEDV, porcine deltacoronavirus (PDCoV), transmissible gastroenteritis virus (TGEV), swine acute diarrhea syndrome coronavirus (SADS), porcine torovirus (PToV), porcine sapelovirus (PSV), group A rotavirus (RVA), group B rotavirus (RVB), and group C rotavirus (RVC) using a commercial one-step qRT–PCR kit (THUNDER-BIRD™ Probe One-step qRT–PCR kit, TOYOBO, Osaka, Japan). The 10 previously reported qRT-PCR assays were performed using the target gene-specific primer and probe sets of each viral pathogen, as described in respective studies (20–26). The reaction composition and conditions were set up according to the manufacturer's instructions.

### 2.3 Virus isolation, purification, and growth kinetics

Vero cells (ATCC CCL-81) were maintained in α-minimum essential medium (α-MEM; Gibco, 12571063) supplemented with 5% fetal bovine serum (FBS; Gibco, A5670701) and 1% antibiotic–antimycotic (Gibco, 15240062) at 37 °C under 5% CO_2_. After mixing 100 μL of sample supernatant with 900 μL of α-MEM containing 5-μg/mL TPCK-treated trypsin (Sigma, 4370285), PEDV-positive intestinal homogenates were inoculated onto confluent Vero cell monolayers in 24-well plates for virus isolation. After incubation, the cultures were serially passaged every 3 days until cytopathic effects (CPEs) characteristic of PEDV infection were observed.

The isolation of PEDV was confirmed via qRT-PCR assay targeting the N gene, as well as using indirect immunofluorescence assay (IFA) showing characteristic CPE. For the IFA, Vero cells infected with the fifth passage (P5) isolate (MOI = 0.01) were fixed with methanol at −20 °C for 10 min, blocked using 1% bovine serum albumin for 1 h, and incubated with a primary monoclonal antibody against the PEDV nucleocapsid protein (3F12, Median Diagnostics, Korea) for 1 h at 37 °C. After washing, the cells were further incubated using a secondary fluorescein isothiocyanate-conjugated anti-mouse IgG antibody (F0257, Sigma-Aldrich) for 1 h at 37 °C. Following secondary-antibody incubation, nuclei were counterstained with DAPI (4′,6-diamidino-2-phenylindole) to visualize the cell nuclei. In addition, fluorescence signals were visualized under a fluorescence microscope.

A PEDV isolate was purified by plaque assay in Vero cells at the third passage. Confluent Vero cells in 6-well plates were infected with serial 10-fold dilutions of the virus, overlaid with α-MEM containing 5-μg/mL TPCK trypsin and 3% agarose (Biosesang, Korea), and incubated at 37 °C for plaque formation. The individual PEDV-specific plaques were then collected and propagated in Vero cells for subsequent passages.

Viral growth kinetics were determined by titration of the purified isolate, using the median tissue-culture infectious dose (TCID50) method. In summary, confluent Vero cells in 96-well plates were infected with 10-fold serial dilutions of virus prepared in α-MEM containing 3-μg/mL trypsin. After 72 h, the CPEs were recorded, and titers were calculated using the Reed and Muench method (27).

### 2.4 Sequence analysis

The complete S gene sequences of three PEDV-positive samples detected from the two pig farms were determined using a published primer set for full-length spike sequencing (28), after which they were sequenced via next-generation sequencing (NGS) on the Illumina platform at a commercial company (BIONICS, Daejeon, Republic of Korea). For whole-genome sequencing of the KPED2021-1 isolate, sequence-independent single-primer amplification was performed as previously described by Wang et al. (28). In summary, viral RNA was reverse-transcribed into first-strand cDNA, using a PrimeScript Double-Strand cDNA Synthesis Kit (Takara, Cat. No. 6110A, Shiga, Japan). Randomly primed cDNA was subsequently amplified with P1 and P2 primers (P1: GAC CAT CTA GCG ACC TCC ACN NNN NNN N, P2: GAC CAT CTA GCG ACC TCC AC TTT TTTTTTT TTTTTTTT TT), followed by Klenow-fragment treatment (New England Biolabs, Ipswich, MA, USA) and PCR amplification with P3 (P3: GAC CAT CTA GCG ACC TCC AC) using the Phusion HF PCR Master Mix (New England Biolabs), according to the manufacturer's instructions. The amplified DNA was subjected to NGS on the Illumina platform at BIONICS BITseq, and raw sequencing reads were quality-trimmed and *de novo*-assembled using Geneious Prime (https://www.geneious.com/).

### 2.5 Multiple alignment and phylogenetic analysis

To ensure diversity in the PEDV phylogenetic analysis, full-genome sequences of 665 PEDV strains were collected from the GenBank database. Based on sequence similarity (99.5% cutoff), the CD-HIT bioinformatics tool was used to cluster identical full-genome sequences from the same country in the same year (29). Finally, 279 full-genome sequences representing the diversity of the PEDV were selected, along with an additional 45 full-genome sequences and 146 S gene sequences of Korean PEDV strains, were used (Supplementary Figure S1). Therefore, phylogenetic analyses were conducted using 325 full-genome sequences and 428 S gene sequences, including one full-genome sequence and the three S gene sequences obtained in this study. The nucleotide and aa sequences were sequence-aligned using MAFFT (30), which is available in Geneious Prime (https://www.geneious.com/). Phylogenetic trees were constructed using the method of RAxML, available in Geneious Prime (https://www.geneious.com/), based on the general time-reversible nucleotide substitution with a gamma distribution model (31). These trees were subjected to bootstrap analysis with 1,000 replicates to determine the percentage reliability values for each internal tree node (32). All phylogenetic trees were visualized using the iTOL phylogenetic tree viewer (33).

### 2.6 Experimental infection and clinical assessment

Ten 5-day-old suckling piglets were obtained from a commercial pig farm with no history of PED outbreaks and confirmed to be seronegative for PEDV antibodies. The piglets were fed a commercial milk replacer thrice daily throughout the experiment. They were then randomly assigned to one of two groups: an infected group (*n* = 5) and an uninfected control group (*n* = 5). The infected group was orally inoculated with 10^5^ TCID_50_/mL of P5 KPED2021-1 isolates diluted in a fresh commercial milk substitute. Meanwhile, the negative control group received an equal volume of sterile cell culture medium diluted in a fresh commercial milk substitute. All animal experiments were approved by the Institutional Animal Care and Use Committee of Jeonbuk National University (approval number: NON2024-073).

The piglets were monitored daily for 7 days post-inoculation (dpi) for clinical signs associated with PEDV infection. Rectal swabs were collected daily, and fecal consistency (FC) was scored according to the following scale: 0 = normal, 1 = pasty, 2 = semi-liquid, and 3 = liquid feces. An FC score ≥2 was considered to indicate diarrhea, as reported in a previous study (34). The body weights were measured at 0 and 7 dpi, and the average daily weight gain (ADWG) was calculated. In addition, survival rates were calculated as the percentage of piglets that survived until the end of the experiment or until euthanasia at 7 dpi for sample collection. Immediately after animals died, intestinal tissues were collected, while tissues from surviving animals were collected after euthanasia at 7 dpi. Euthanasia was performed via electrocution following an intramuscular injection of azaperone (40 mg/mL, Stresnil^®^, Elanco Animal Health) at a dose of approximately 2.2 mg/kg body weight. The pigs were allowed to reach adequate sedation of approximately 10–15 min post-injection. Once adequate sedation was confirmed, electrocution was performed using 60 Hz alternating current applied across the brain and heart for 5–10 s to ensure immediate loss of consciousness. Exsanguination was performed immediately following electrocution to complete the euthanasia process. The viral RNA loads in rectal swabs and intestinal tissues were quantified using the PEDV-specific qRT-PCR assay described in Section 2.2.

### 2.7 Histological and immunohistochemical analyses

Small intestinal samples were collected at necropsy from pigs in both the infected and negative control groups. The tissues were fixed in 10% neutral buffered formalin, embedded in paraffin, and sectioned for histopathological examination. Hematoxylin and eosin (H&E) staining was then performed for histological analysis, to confirm whether morphological changes occurred in the villi and crypts in the small intestine associated with PEDV infection. Using a mouse monoclonal antibody specific to the PEDV-N protein (3F12, Median Diagnostics, Chuncheon, Republic of Korea), immunohistochemistry (IHC) analysis was conducted to detect and localize PEDV antigens within the intestinal tissues. Both H&E staining and IHC analyses were performed by Oben Bio (Suwon, Republic of Korea), a specialized laboratory service provider.

### 2.8 Statistical analysis

All statistical analyses were performed using GraphPad Prism 10 (GraphPad, San Diego, CA, USA). The fecal score, ADWG, and viral loads from rectal swabs and intestinal tissues were analyzed using a non-parametric *t*-test (Mann–Whitney U test), to compare the infected and control groups at each time point. This non-parametric test was selected because of the small sample size and the potential non-normal distribution of the data. Data are presented as the mean ± standard deviation, with *p*-values (*p*) < 0.05 considered statistically significant (35).

## 3 Results

### 3.1 Identification and isolation of KPEDV2021-1 strains

**Figure 1:**
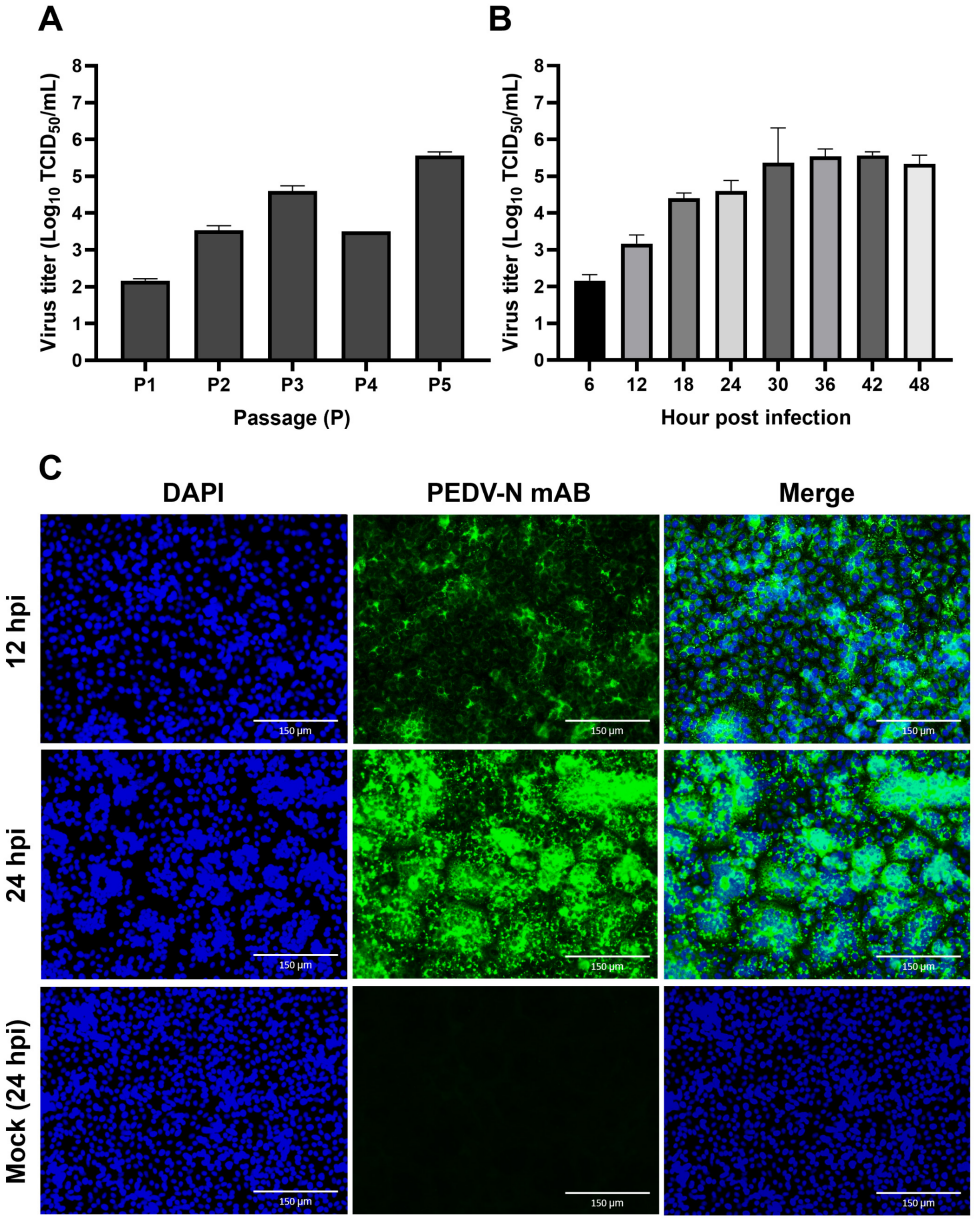
Growth kinetics and immunofluorescence analysis of the KPED2021-1 strain in Vero cells. **(A)** Virus titers of the KPED2021-1 strain during serial passages in Vero cells. The virus titers (Log_10_ TCID_50_/mL) were determined from Passages 1 to 5. **(B)** Growth curve of P5 KPED2021-1 strain in Vero cells. Cells were infected at an MOI of 0.01, and virus titers were determined at 6-h intervals from 6 to 48 h post-infection. **(C)** Immunofluorescence analysis of PEDV-N protein expression in infected Vero cells. Cells were infected with the P5 KPED2021-1 strain and fixed at 12 and 24 h post-infection (hpi). Mock-infected cells (24 hpi) served as a negative control. Cells were stained with DAPI (blue) for nuclei and anti-PEDV-N monoclonal antibody (green) for viral-protein expression. Scale bars = 150 μm. Data represents the mean ± standard deviation from three independent experiments.

Based on N gene-specific qRT-PCR, all three intestinal samples collected from the two affected farms tested positive for PEDV, with the Ct values of the Farm A sample (2021-1) being 14.02, and those from the Farm B samples (2022-1 and 2202) being 14.56 and 17.22, respectively. However, eight other enteric viruses (PDCoV, TGEV, SADS-CoV, PToV, PSV, RVA, RVB, and RVC) tested negatively in each virus-specific qRT-PCR assay. For virus isolation, clarified intestinal homogenates were inoculated onto Vero cells and serially blind-passaged. PEDV-associated CPEs, characterized by cell fusion and syncytium formation, were first observed at the second passage in cultures inoculated with the 2021-1 sample (Supplementary Figure S2), whereas no CPE developed in cells inoculated with the 2022-1 or 2022-2 samples, even after five blind passages. The successfully isolated strain was designated KPED2021-1. Subsequently, the isolate was plaque-purified at P3 and propagated in Vero cells. Replication kinetics across sequential passages demonstrated that KPED2021-1 yielded titers ranging from 10^2.2^ to 10^5.6^ TCID_50_/mL between P1 and P5 (Figure 1A). To further characterize the viral replication, a growth curve was generated using the P5 virus stock. The viral titers increased from 10^2.1^ TCID_50_/mL at 6 hpi to a maximum of 10^5.7^ TCID_50_/mL at 42 hpi, followed by a slight decline at 48 hpi, coinciding with cell detachment due to extensive CPEs (Figure 1B). Moreover, KPED2021-1 antigen expression was confirmed via IFA. The specific green fluorescence in PEDV-infected cells was detectable at 12 hpi, whereas extensive PEDV-infected cells were observed at 24 hpi, confirming that this study successfully isolated the KPED2021-1 strain (Figure 1C).

### 3.2 Genetic characterization of the S gene of KPED2021-1 and KP strains

Complete S gene sequences were successfully obtained from all three PEDV-positive intestinal samples and deposited in GenBank as KPED2021-1 from Farm A (accession number OK465397), and KP2022-1 and KP2022-2 from Farm B (accession numbers OR125555 and OR125556, respectively). Comparative sequence analysis revealed that the S gene sequences of the three strains were 100% identical at the nucleotide levels. Accordingly, genetic analyses were conducted using the KPED2021-1 strain as a representative isolate.

To assess the genetic relatedness of the KPED2021-1 strain, closely related strains were identified using the Basic Local Alignment Search Tool (BLAST, https://blast.ncbi.nlm.nih.gov). The S gene of KPED2021-1 showed high nucleotide sequence identity (99.28%−99.71%) with PEDV strains reported in China between 2016 and 2019. Contrastingly, lower similarity was observed with previously reported circulating Korean G2 strains (Table 1). Among the Chinese strains, CH/JLDH/2016 and HM2017 exhibited the highest sequence identity, at 99.71% (12 nucleotide differences) and 99.45% (23 nucleotide differences), respectively.

**Table 1:** Summary of the basic local alignment search tool results for the spike gene of the KPED2021-1 and KP strains.

Order	Strain name	Query cover (%)	Identity (%)	Country	Year	GenBank No.
1	CH/JLDH/2016	100	99.71	China	2016	MF346935
2	CH/SCCZ/2017	100	99.66	China	2017	MH053419
3	HBHG1	100	99.54	China	2016	KY775045
4	GDhz18	100	99.49	China	2018	MN368716
5	SDbz18	100	99.49	China	2018	MN368709
6	HM2017	100	99.45	China	2017	MK690502
7	HLJYTX-2018	100	99.42	China	2018	MT294128
8	SWUN19/CH/SCZY	100	99.40	China	2019	MK592415
9	CH/HBXY/2018	100	99.37	China	2018	MH816969
10	CH/SCXC/2018	100	99.28	China	2018	MK598821

**Figure 2:**
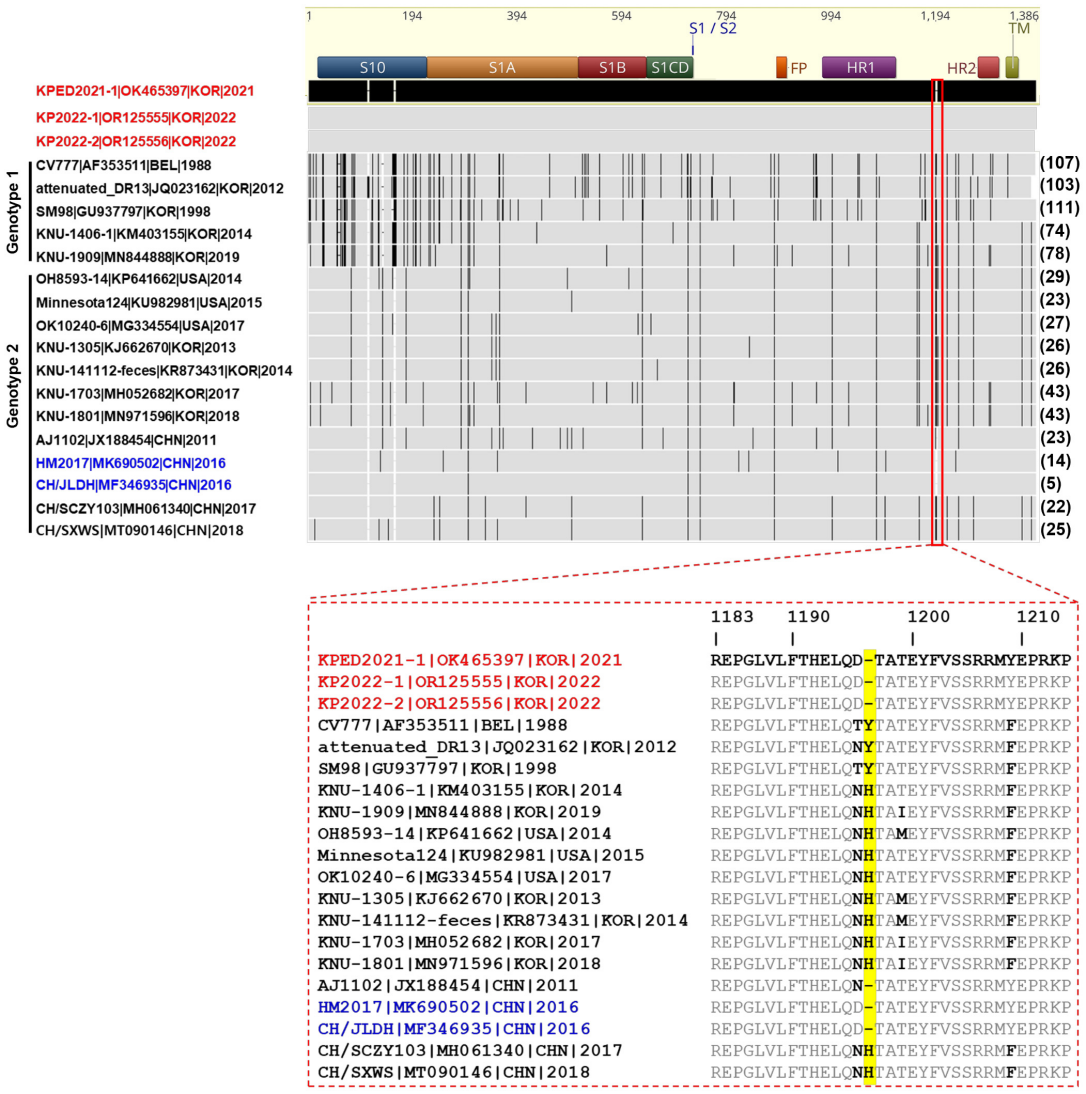
Alignment of amino acid (aa) sequences of the spike protein of the KPED2021-1 and KP strains with global porcine epidemic diarrhea virus strains. The top diagram map to the organization of the Spike (S) protein, including the putative cleavage site between the S1 and S2 subunits, S1 domains 0, A, B, and CD, fusion peptide (FP), heptad repeat regions (HR1 and HR2), and transmembrane domain (TM). The red strain name indicates PEDV isolates in this study. The blue strains are the Chinese strain most similar to the KPEDV2021-1 strain. Gray areas represent identical amino acid (aa) sequences to KP strains, and each vertical black bar represents one aa sequence divergent from KP strains. Numbers in parentheses on the right indicate the aa differences compared with KPED2021-1 and KP strains. The 1183–1214 aa position of the S protein is enlarged as a red rectangle, and the aa different from KP strains are displayed in bold. The 1197 aa position of the S protein containing aa deletion (shown as black dashes) in KPED2021-1 and KP strains and Chinese PEDV strains is marked with a red background color.

Based on multiple-sequence alignment, aa differences in the KPED2021-1 S protein were evaluated in comparison with representative global PEDV strains classified as G1 and G2. A comparison with G1 PEDV strains revealed ranging from 74 to 111 substantial aa differences across the full-length S protein (Figure 2), and Chinese strains CH/JLDH/2016 and HM2017 differed by a small number of aa residues (14 and 5 aa, respectively), whereas substantially larger numbers of aa differences were observed when comparing with Korean G2 strains (KNU-1305, KNU-141112-feces, KNU-1703, and KNU-1801), with 23–43 aa differences identified in the S protein. These quantitative differences highlight that KPED2021-1 is genetically distinct from classical G1 PEDV strains and from Korean G2 strains, while maintaining closer aa similarity to Chinese PEDV variants at the S protein (Figure 2). A comparative analysis of the deduced aa sequences revealed that KPED2021-1 and the two KP strains shared a unique aa deletion in the S protein, corresponding to position 1197, which has been reported exclusively in a subset of Chinese PEDV strains but has not been identified in Korean G2b strains (17, 18, 36) (Figure 2).

**Figure 3:**
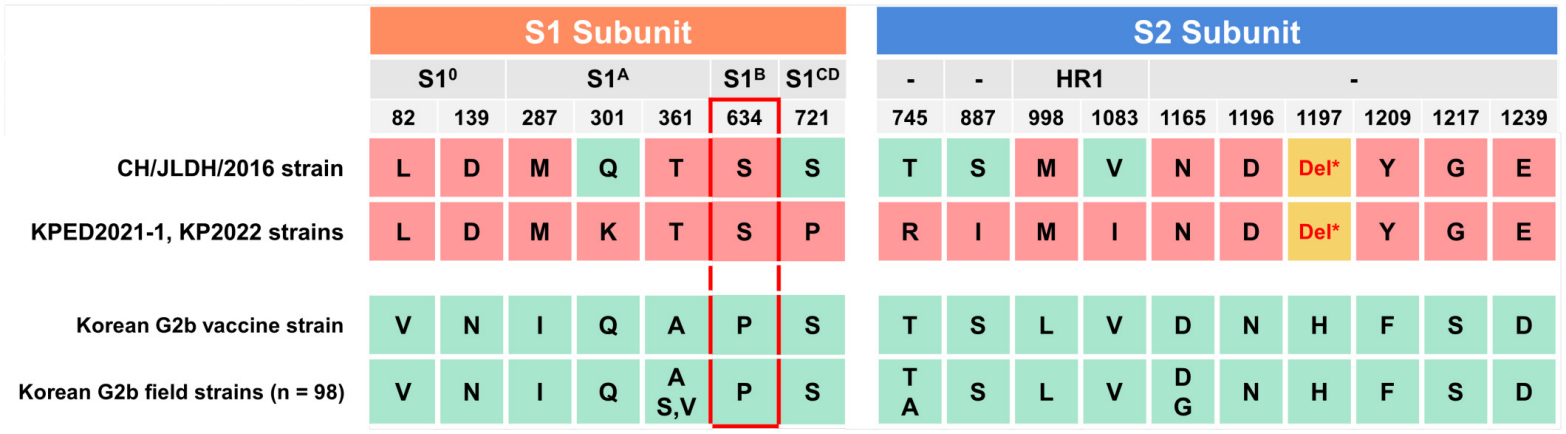
Comparison of significant shared amino acid (aa) variations in the spike protein among the KPED2021-1 and KP strains, the Chinese strain CH/JLDH/2016, the Korean G2b vaccine strain, and 98 Korean G2b field strains. The spike protein is divided into S1 and S2 subunits, with specific domains indicated (S10, S1A, S1B, S1CD, and HR1). Dashes (-) indicate regions with undefined functional domains. Del* is an amino acid deletion (Δ). Numbers represent amino acid positions. Amino acid differences are highlighted in red (distinct variations), green (conserved residues), and yellow (deletion). The P634S substitution within the core neutralizing epitope (COE, indicated by red dotted box) and H1197Δ in the S2 region represent notable variations in the KPED2021-1 and KP strains compared to Korean G2b field strains. The consensus sequence was derived from 98 Korean G2b strains, with predominant amino acids shown above and minor variants indicated below (e.g., A/S, V indicating Alanine as predominant with serine and valine as minor variant at the specified position).

To identify characteristic aa substitutions in the KPED2021-1 isolate and KP2022 strains (KP2022-1 and KP2022-2) reported in this study, compared with previously reported Korean G2b PEDV strains, a domain-based comparison was performed. The domain-based comparison of the S protein was performed by subdividing the protein into S1 and S2 subunits and their respective functional domains, with reference to the subdivision reported in a previous study (37, 38). The KPED2021-1 isolate, KP2022 strains, one Chinese PEDV CH/JLDH/2016 isolate similar to KPED2021-1, one Korean live attenuated vaccine strain (KNU-141112_DEL5/ORF3), and 98 Korean G2b field strains identified among 146 collected Korean PEDV S gene sequences were included in the domain-based comparison of the S protein. Within the S1 subunit, residues located in the S1^0^ domain (82L and 139D) and the S1^A^ domain (287M and 361T) of KPED2021-1 were identical aa residues to those of CH/JLDH/2016 but differed from those conserved among Korean G2b strains. Notably, residue 634 within the core neutralizing epitope (COE) of the S1^B^ domain was conserved as serine in KPED2021-1 and CH/JLDH/2016, whereas proline was consistently observed at this position in 98 Korean G2b strains (Figure 3). An analysis of the S2 subunit further demonstrated multiple aa variations distinguishing KPED2021-1 from Korean G2b strains. Within the heptad repeat 1 (HR1) region, residue 998 was conserved as methionine (M) in KPED2021-1 and CH/JLDH/2016 but differed from the leucine (L) observed in Korean G2b strains. In the C-terminal region of the S protein, six residues (1165N, 1196D, 1197deletion, 1209Y, 1217G, and 1239E) were identical between KPED2021-1 and CH/JLDH/2016 yet distinct from those conserved in the Korean G2b vaccine strain and 98 Korean G2b field strains. However, the KPEDV2021-1 and KP2022 strains also exhibited unique characteristics, with five substitutions (301K, 721P, 745R, 887I, and 1083I) that differed from both the CH/JLDH/2016 and Korean G2b strains (Figure 3), suggesting the emergence of a new variant that evolved from a CH/JLDH/2016-like virus rather than from the Korean G2b strains. These findings indicate that the KPEDV2021-1 and KP2022 strains represent PEDV variants that are more closely related to Chinese strains than to Korean strains but that maintain unique substitutions that distinguish them from both reference strains.

### 3.3 Full genome sequencing and molecular characterization of KPED2021-1

**Figure 4:**
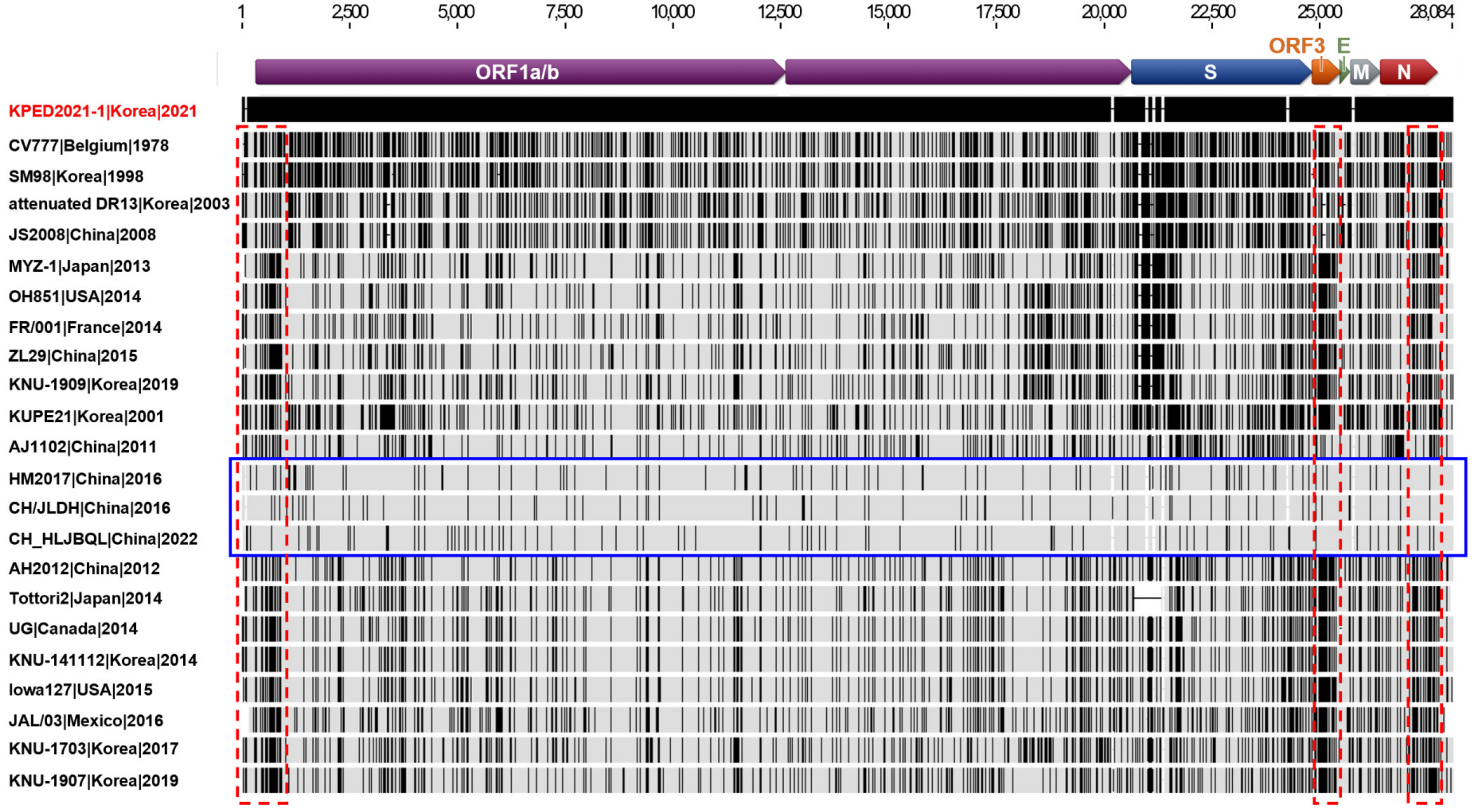
Nucleotide Sequence alignments and similarity plot analysis of the full genome sequences of the KPED2021-1 isolate and global porcine epidemic diarrhea virus strains. The full-genome sequences of 22 PEDV strains representing each genotype were aligned based on the KPED2021-1 isolate. Gray areas represent identical nucleotide (nt) sequences to the KPED2021-1 isolate, and each vertical black bar represents nt sequence divergence from the KPED2021-1 isolate. The blue rectangle indicates Chinese G2 PEDV strains that have high homology with the KPED2021-1 isolate. Regions exhibiting increased nucleotide divergence in comparisons excluding these Chinese strains are indicated by red dotted rectangles.

The complete genome sequence of the KPED2021-1 isolate was sequenced and compared with global PEDV strains (Table 2). The KPED2021-1 genome (GenBank accession number OK465397) exhibited high nucleotide sequence identity (99.6%−99.7%) with Chinese PEDV strains CH/JLDH/2016, HM2017, and CH_HLJBQL, which have been reported as G2 variants circulating in northeastern China, including Jilin and Heilongjiang provinces (17, 18, 39). In contrast, a comparison with PEDV strains circulating in South Korea between 2013 and 2019 revealed lower nucleotide sequence identity (97.9%−98.7%). Consistent with these identity results, full-genome multiple-sequence alignment demonstrated that KPED2021-1 shared extensive nucleotide conservation with the Chinese PEDV strains, whereas more nucleotide mismatches were observed when KPED2021-1 was compared with Korean PEDV strains and other global strains (Figure 4). Although nucleotide differences were detected throughout the genome, sequence variation in KPED2021-1 was not evenly distributed. Notably, when compared with Korean G2 PEDV strains, a higher density of nucleotide mismatches was observed in the NSP1-coding region within ORF1a, as well as in ORF3 and the nucleocapsid (N) gene, distinguishing KPED2021-1 from previously circulating Korean PEDV strains (Figure 4).

**Table 2:** Comparison of the complete genomic sequences between the KPED2021-1 isolate and global porcine epidemic diarrhea virus strains.

Strain name	Country	Year	Genotype	Nucleotide identity (%)	GenBank No.
CV777^*^	Belgium	1988	G1a	96.6	AF353511
SM98^a^	Korea	1998	G1a	96.1	GU937797
KUPE21	Korea	2001	G1a	97.9	MF737355
Attenuated DR13^a^	Korea	2009	G1b	96.7	JQ023161
KNU-1305	Korea	2013	G2b	98.7	KJ662670
KNU-141112	Korea	2014	G2b	98.7	KR873431
KNU-141112 S DEL5/ORF3^a^	Korea	2017	G2b	98.4	KY825243
KNU-1703	Korea	2017	G2b	98.3	MH052682
KNU-1904	Korea	2019	G2b	98.3	MN971595
KNU-1909	Korea	2019	G1c	97.9	MN844888
JS2008	China	2008	G1a	96.8	KC210146
AJ1102	China	2011	G2c	98.7	JX188454
AH2012	China	2012	G2b	98.6	KC210145
ZL29	China	2015	G1c	98.1	KU847996
HM2017	China	2016	G2c	99.6	MK690502
CH/JLDH	China	2016	G2c	99.7	MF346935
CH_HLJBQL	China	2022	G2c	99.7	OM914738
OH851	United States	2014	G1c	98.3	KJ399978
Iowa127	United States	2015	G1c	98.6	KU982969
MZY-1	Japan	2013	G1c	98.3	LC063846
Tottori2	Japan	2014	G2b	96.5	LC022792
FR/001	France	2014	G2b	98.2	KR011756
UG	Canada	2014	G2b	98.6	MZ803010
JAL/03	Mexico	2016	G2b	98.4	MH004413

^*^CV777 is the prototype strain of PEDV.

^a^Korean commercial vaccine strains.

### 3.4 Phylogenetic analysis

**Figure 5:**
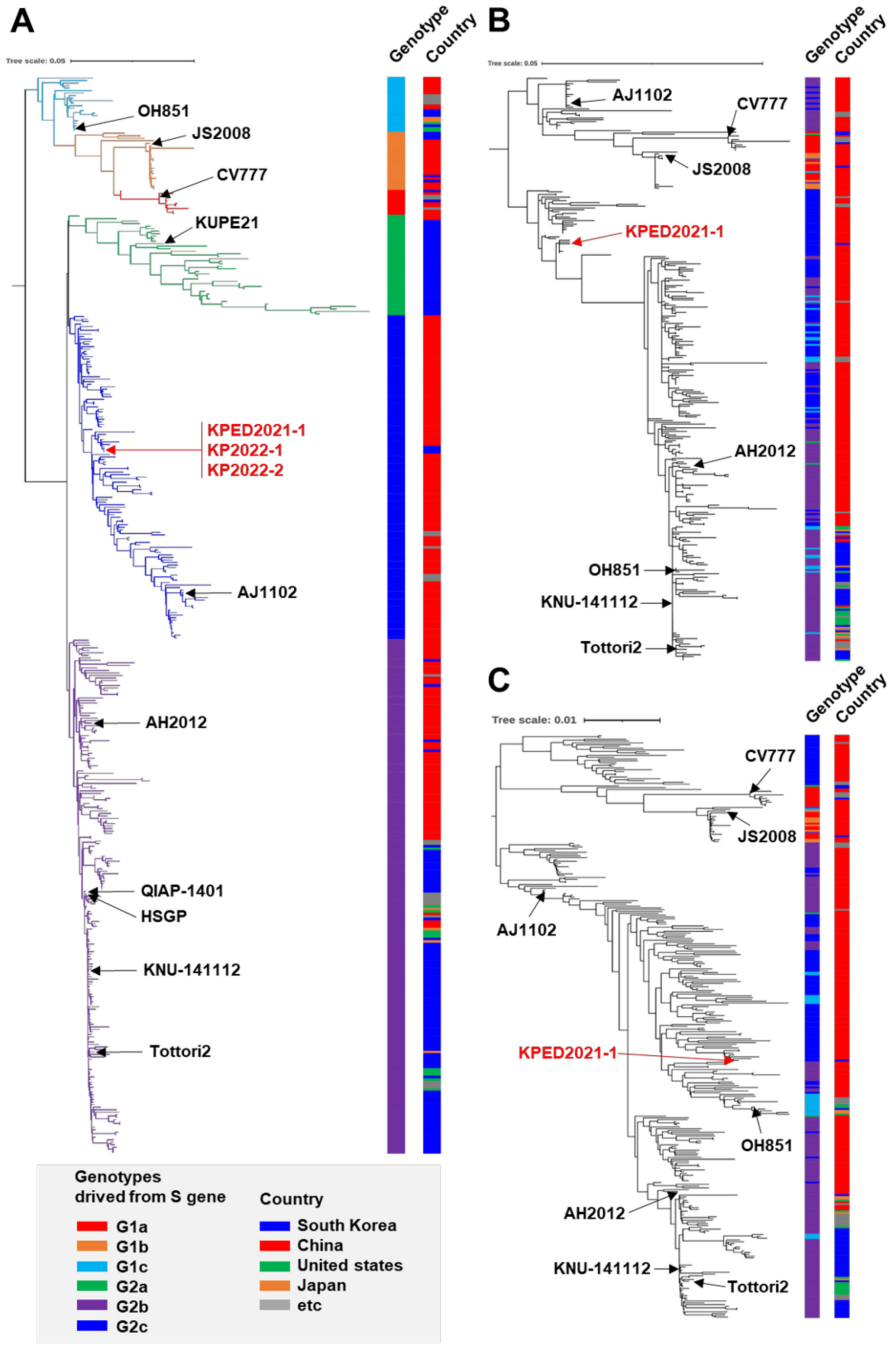
Phylogenetic analysis of the S gene, N gene, and full genome of KP strains with global porcine epidemic diarrhea virus strains. Midpoint-rooted maximum-likelihood phylogenetic trees constructed using the GAMMA GTR model based on the **(A)** S gene (*n* = 428), **(B)** N gene (*n* = 325), and **(C)** full genome (*n* = 325) of global PEDV strains. Black arrows indicate the prototype and reference PEDV strains. Red arrows indicate the KP strains identified in this study. Genotypes derived from the S gene, along with the countries of the strains, are listed on the right side of each strain with the indicated color. Scale bars indicate nucleotide substitutions per site.

Phylogenetic analysis based on the S gene showed that global PEDV strains were classified into two major genotypes, G1 and G2, which were further classified into six sub-genotypes, consistent with a previous proposed classification report (5). The following reference strains representing each sub-genotype were included in the analysis: CV777 (G1a); SM98 and attenuated DR13 (G1b); OH851 (G1c); KUPE21 (G2a); AH2012, Colorado_2013, and QIAP-1401 (G2b); and AJ1102, CH/JLDH/2016, and HM2017 (G2c). In the S-gene-based phylogenetic tree, all previously reported Korean PEDV strains clustered within the G2b. Contrastingly, KPED2021-1, together with KP2022-1 and KP2022-2, clustered within the G2c and grouped with PEDV strains originating from China (Figure 5A). G2c exhibited a distinct geographical distribution pattern, with isolates from China being the predominant population within this classification. In addition, the group included three strains, each from Vietnam and Thailand, demonstrating limited geographical diversity within this cluster (Figure 5A). The KP strains were positioned within this Chinese-dominant G2c cluster and were clearly separated from the Korean G2b cluster in the S gene phylogeny (Figure 5A). Phylogenetic analyses based on N gene (Figure 5B) and whole-genome (Figure 5C) sequences revealed a complex genotype topology that did not show the distinct genotype-specific clustering observed in the S gene analysis. Unlike the S-gene-based phylogenetic tree, which differentiated six sub-genotypes (G1a–G1c and G2a–G2c), the N gene and whole-genome analyses showed more divergent branching patterns with less defined genotype boundaries. Nevertheless, the KPED2021-1 and KP strains maintained their close phylogenetic relationship with Chinese PEDV strains across all three genomic analyses (S gene, N gene, and whole genome). This clustering pattern consistent with Chinese strains, observed across different genomic regions despite varying tree topologies, provides evidence of the evolutionary relationship between these newly identified Korean strains and Chinese viral populations.

### 3.5 Pathogenicity and virulence of the PEDV strain KPED2021-1

The KPED2021-1 isolate showed high pathogenicity in a 7-day challenge experiment. Ten 5-day-old suckling piglets confirmed to be seronegative for PEDV were randomly divided into two groups: an infected group (*n* = 5) and an uninfected control group (*n* = 5). The infected group was orally inoculated with a commercial milk substitute containing 5 log_10_ TCID_50_ of the P5 KPED2021-1 isolate, while the control group received a commercial milk substitute containing sterile culture medium as a mock inoculation.

**Figure 6:**
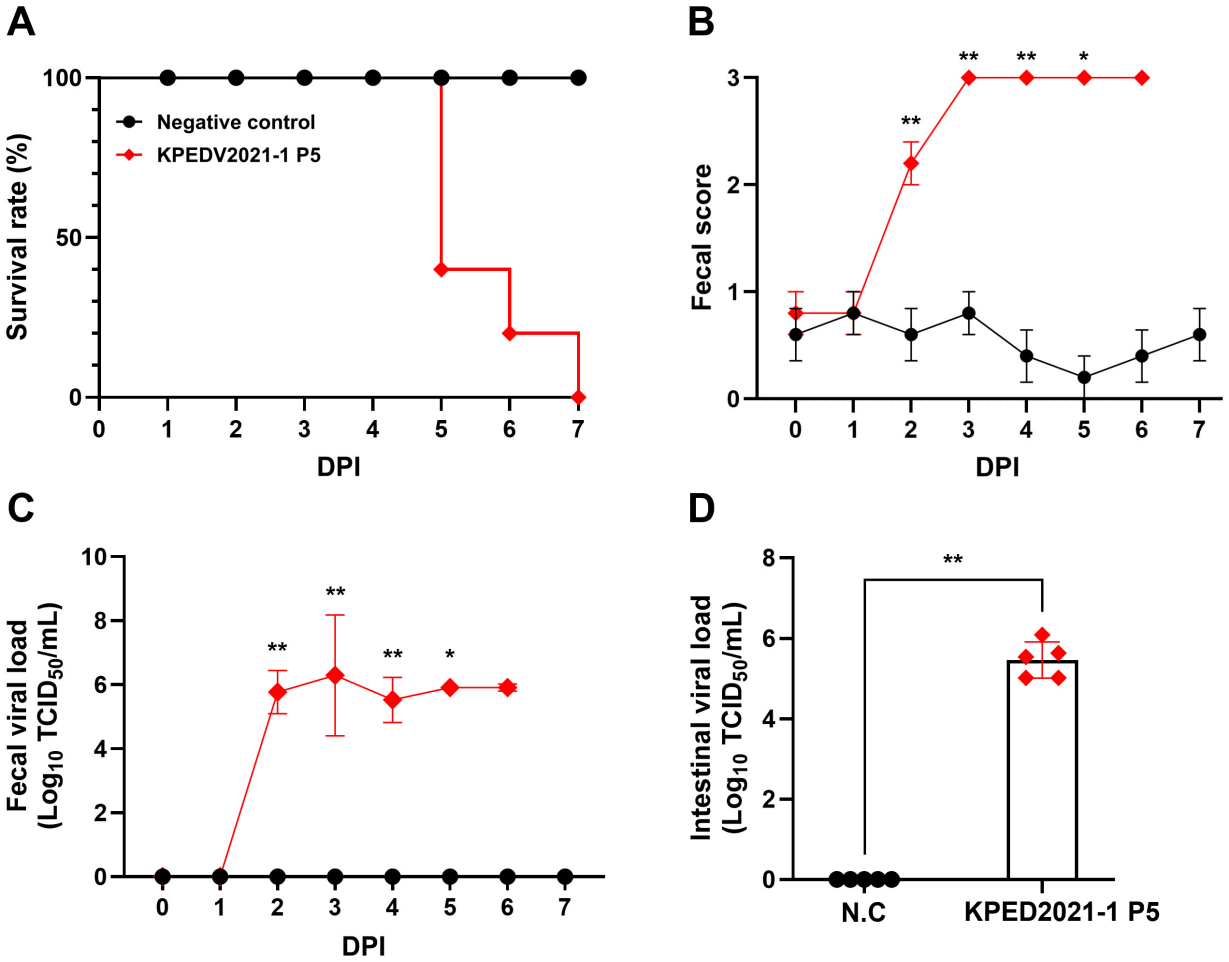
Virulence assessment of the KPED2021-1 strain in experimentally infected piglets. **(A)** Survival rates of piglets after KPED2021-1 Passage 5 (P5) infection. **(B)** Clinical fecal scores in mock and KPED2021-1-P5-infected piglets. **(C)** Fecal viral shedding kinetics determined using RT-qPCR for PEDV. **(D)** Intestinal viral loads at 7 dpi, measured using RT-qPCR for PEDV. Viral-load data are expressed as Log_10_ TCID_50_/mL. Fecal scores and viral load values were censored at the time of death. Statistical analyses and graphical presentations at each time point included only observations from animals that were alive at that time. Fecal score and viral-load values represent means ± standard deviation. **, *p* < 0.01, *, *p* < 0.05. N.C., negative control.

Following inoculation, three piglets (P5-1, P5-2, and P5-3) in the infected group died at 5 dpi, and one piglet (P5-5) died at 6 dpi, followed by the remaining piglet at 7 dpi. This resulted in a total mortality rate of 100% by the end of the experiment. In contrast, all negative control piglets maintained 100% survival throughout the experiment (Figure 6A, Supplementary Table S1). Clinical symptoms manifested rapidly in the infected group, with fecal scores increasing significantly from 2 dpi (*p* < 0.01). The severity of diarrhea progressively worsened, reaching scores of 3 by 3 dpi and remaining high until death, whereas the control group consistently showed *normal* fecal scores (0–1) throughout the study (Figure 6B). For the KPED2021-1-infected group, viral shedding in fecal swabs was first detected at 2 dpi, peaked at approximately 6 log_10_ TCID_50_/mL at 3 dpi, and maintained high viral loads (> 5.5 log10 TCID_50_/mL) until death. Moreover, the intestinal viral-load analysis from the infected group revealed significant viral presence (approximate average of 5.5 ± 0.4 log_10_ TCID_50_/mL; *p* < 0.01), whereas no virus was detected in fecal swabs or small intestinal samples from the control group (Figures 6C,D).

The KPED2021-1-infected group exhibited weight loss, but negative control piglets showed consistent weight gain, with the ADWG value ranging from 15.71 to 84.29 g/day (mean: 42.43 ± 28.4 g/day). Meanwhile, the ADWG values in the infected group ranged from −69.29 to −455 g/day (mean: −304.2 ± 134.3 g/day), with initial weights of 1.35–2.28 kg, which declined to 0.95–1.74 kg by the time of death. These comprehensive results demonstrate that the KPED2021-1 strain is virulent, causing severe clinical manifestations including acute diarrhea, substantial weight loss, and 100% mortality within 7 dpi.

**Figure 7:**
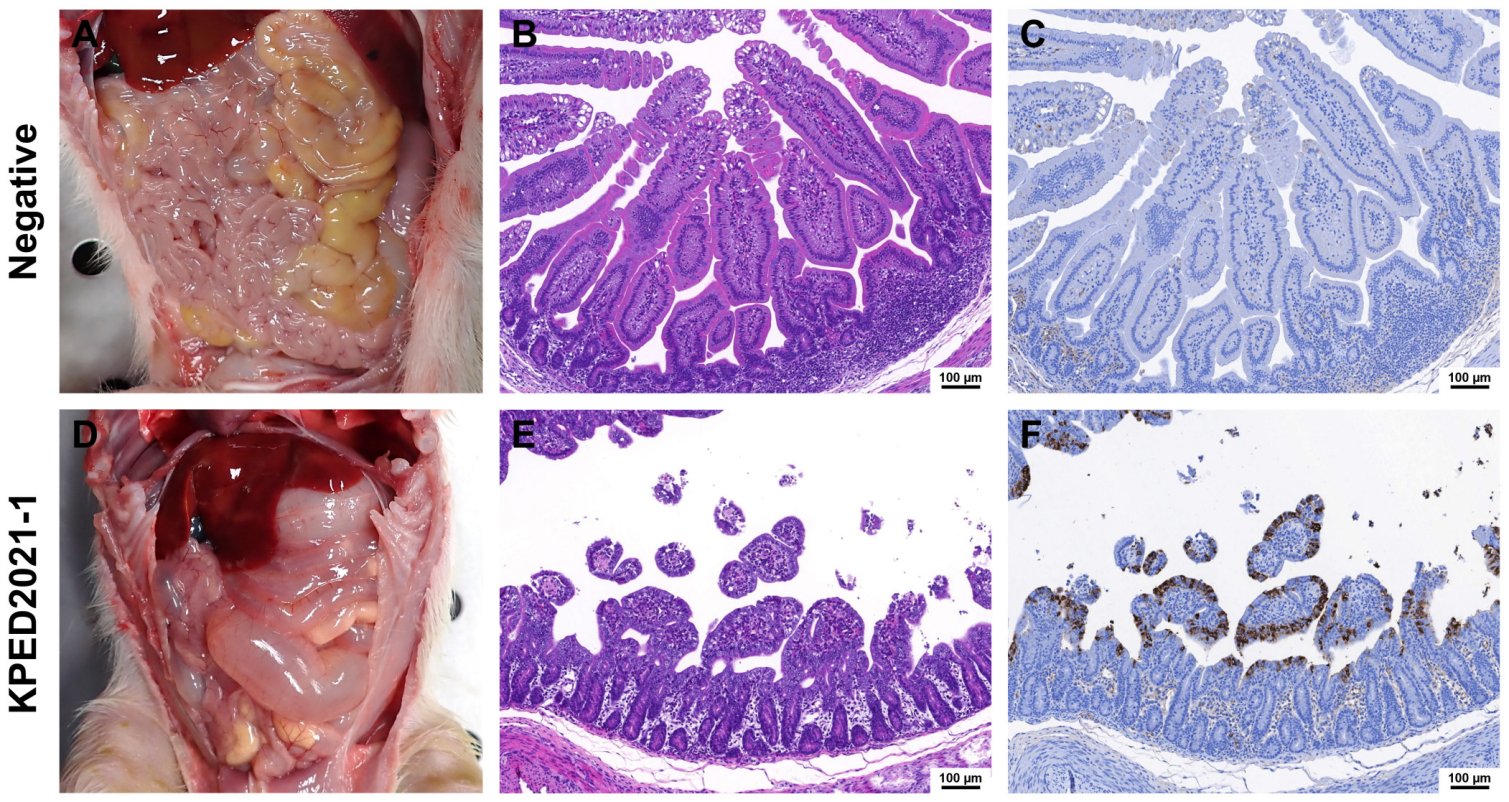
Evaluation of pathogenicity of KPED2021-1 strain in piglet small intestines at 7 days post-inoculation. **(A, D)** Gross lesions of the small intestine of a KPED2021-1-inoculated piglet and a negative piglet after autopsy. **(B, E)** Hematoxylin and eosin staining of small intestine sections of the KPED2021-1-inoculated piglet and the negative piglet. **(C, F)** Immunohistochemistry analysis for PEDV antigen detection. Scale bars = 100 μm.

Histopathological and immunohistochemical analyses were performed to evaluate the pathogenicity of the KPED2021-1 strain in the small intestines of affected piglets, facilitating the further characterization of the pathogenic effects. Based on H&E staining, the negative control group showed normal intestinal morphology with long, intact villi (Figure 7B), and no PEDV antigen was detected by IHC (Figure 7C). Contrastingly, the KPED2021-1-infected group exhibited severe histopathological changes characterized by extensive villous atrophy, fusion, and destruction of the intestinal epithelium (Figure 7E). These microscopic lesions corresponded to the gross pathological changes observed in the small intestines, which showed thin and transparent intestinal walls with distention (Figure 7D) compared with their normal appearance in the control group (Figure 7A). In addition, IHC analysis revealed strong positive staining for PEDV antigens (brown color) in the cytoplasm of villous enterocytes in the KPED2021-1-infected group (Figure 7F), whereas no viral antigens were detected in the negative control group (Figure 7C). The distribution of PEDV antigens coincided with the areas showing severe histopathological lesions, indicating active viral replication in the damaged intestinal epithelium. These histopathological and immunohistochemical findings show that the KPED2021-1 strain causes severe intestinal damage characterized by villous atrophy and extensive viral replication in the small intestines of infected piglets.

## 4 Discussion

Under field conditions, PEDV continues to evolve, and its high genetic diversity persistently threatens disease control in swine populations. The global spread of highly virulent G2b PEDV strains during the 2013–2014 pandemic highlighted the potential for the rapid emergence and transboundary dissemination of novel variants (6, 14). Therefore, to manage this ongoing threat to the global swine industry, careful monitoring to track the emergence of a new variant and prompt the development of a vaccine suitable for preventing the new variant are crucial.

In the present study, PEDV was detected in two epidemiologically unrelated pig farms experiencing severe diarrhea and nearly 100% mortality in suckling piglets despite regular vaccination with commercial G2b-based vaccines in their sow herds. Three clinical samples obtained from these farms yielded identical full-length S gene sequences, and one strain, designated KPED2021-1, was successfully isolated in Vero cells. The isolate was confirmed based on typical CPEs and using both N gene-specific qRT-PCR and the immunofluorescence detection of PEDV.

Given the main role of the S glycoprotein in coronavirus attachment, entry, and antigenicity, aa variations within functionally defined regions of the S protein provide critical insights into strain-specific biological properties (40). The PEDV S protein is composed of two functional subunits, namely the S1 subunit, which mediates receptor recognition and has major neutralizing epitopes, and the S2 subunit, which drives membrane fusion during viral entry (40). According to the comparative analysis of the S gene, KPED2021-1 exhibits more than 99% sequence homology with Chinese PEDV strains reported between 2016 and 2019 and shares several genetic characteristics with these strains. Accordingly, a comparative analysis of conserved aa residues between the Korean G2b vaccine and 98 field strains, as well as the KPED2021-1 and KP strains, identified a total of 16 aa substitutions and one deletion across the S protein, including seven substitutions in the S1 subunit and nine substitutions, together with one deletion, in the S2 subunit. Among these substitutions, 11 substitutions and one characteristic deletion were identical to those observed in Chinese PEDV strains. Notably, a distinctive aa deletion at position 1197, which has not been identified in previously reported Korean G2b strains, was detected in KPED2021-1. Although this deletion represents a unique genetic feature of KPED2021-1, its functional significance within the PEDV S protein remains unknown and warrants further investigation.

Through domain-based analysis, several aa substitutions specific to KPED2021-1 were identified. These substitutions were most similar to those observed in Chinese PEDV variant strains and were absent from previously reported Korean G2b field strains. Mapping these substitutions to the S protein revealed that they were located within multiple functionally defined regions. In the S1 subunit, two substitutions were identified in the S1^0^ domain, three in the S1^A^ domain, and one each in the S1^B^ and S1^CD^ domains (38, 41). In the S2 subunit, two substitutions were located within the HR1 region, which participates in membrane fusion (37, 40). Notably, a P634S substitution was detected within the CO-26K equivalent epitope (COE) of the S1 subunit (42), and a deletion at position 1197 (H1197Δ) was observed in the S2 subunit. Both features were absent from previously circulating Korean G2b strains. Taken together, the overall pattern of S protein variation in KPED2021-1 revealed substantial overlap with that of the Chinese variant strain CH/JLDH/2016, which indicated a shared evolutionary background distinct from previously circulating Korean G2b strains. Meanwhile, several substitutions were uniquely observed in KPED2021-1 and KP strains, suggesting additional diversification following divergence from a Chinese-like ancestor. Although this study did not directly evaluate the functional consequences of individual substitutions, these findings highlight the importance of continued surveillance and further investigation to clarify their implications for viral evolution, vaccine efficacy, and disease control strategies.

Whole-genome analysis further supported the Chinese-like genetic background of KPED2021-1. The isolate exhibited higher nucleotide sequence identity with Chinese PEDV strains than with circulating Korean strains, and genome-wide alignment patterns showed extensive conservation relative to Chinese strains but greater divergence relative to Korean and other global strains. Although nucleotide substitutions were detected throughout the genome, nucleotide differences relative to Korean strains were preferentially enriched in the NSP1-coding region within ORF1a, as well as in ORF3 and the N gene. These genes have been shown to play critical roles in viral replication dynamics and the modulation of host immune responses in PEDV infections. The NSP1 protein suppresses host gene expression and interferes with interferon responses (43, 44), while ORF3 contributes to viral pathogenesis by delaying apoptosis and suppressing inflammatory responses (45, 46). The N protein is essential for viral genome replication, and it modulates cell cycle progression and interferon production (47, 48). Therefore, continuous genome-based surveillance of genes associated with viral replication and virulence is essential to elucidate the evolutionary trajectories of PEDV strains and their implications for disease epidemiology.

Phylogenetic analysis based on S gene sequences showed that these sequences could be classified into two main genotypes (G1 and G2), each of which could be further divided into three sub-genotypes. A previous study conducted on Korean PEDV strains classified G2 into two main genotypes: G2a, which primarily occurred in Asia before 2013, and G2b, which was similar to the epidemic PEDV strains found in the US in 2013 (6). However, recent studies have proposed a more refined classification by introducing a third group and have subdivided G2 into three distinct sub-genotypes: G2a, G2b, and G2c (4, 5, 49). Under this S gene-oriented phylogenetic context, recently reported circulating Korean PEDV strains clustered within the G2b lineage, whereas KPED2021-1 and the related KP2022 strains included a distinct clade with PEDV strains predominantly originating from emerging Chinese highly pathogenic strains (17, 18).

In contrast, phylogenetic trees reconstructed using the N gene and complete genome sequences did not reproduce the same genotype-specific clustering patterns observed in the S gene-based analysis but instead displayed more complex and partially incongruent topologies. These differences likely reflected evolutionary constraints among viral genes, with the S gene undergoing stronger selective pressures related to host adaptation and immune escape, while other genomic regions remained more conserved. Importantly, despite these differences in tree topology, KPED2021-1 and the KP strains consistently exhibited closer phylogenetic proximity to Chinese PEDV strains than to recently circulating Korean strains. The placement of KPED2021-1 within a Chinese variant clade supports the interpretation that this strain indicates a newly introduced PEDV lineage in South Korea rather than a derivative of the domestic G2b lineage.

The pathogenicity assessment of KPED2021-1 demonstrated that this isolate is virulent in experimentally infected suckling piglets. In the KPED2021-1-infected group, 100% mortality was observed within 7 days post-infection (dpi), with most piglets succumbing by 5 and 6 dpi. Furthermore, infected piglets showed acute diarrhea, with fecal scores indicating maximum severity by 4 dpi, as well as substantial weight loss. Fecal viral shedding peaked at approximately 6 log_10_ TCID_50_/mL by 3 dpi and remained high until death. The ADWG in the infected group ranged from −69.29 to −455 g/day, with infected piglets exhibiting more than 20% weight loss, contrasting with the weight gain observed in the negative control group. Moreover, histopathological analysis revealed severe villous atrophy, fusion, and destruction of the intestinal epithelium. These microscopic lesions corresponded with gross pathological findings, including thin and translucent intestinal walls. IHC analysis further confirmed an extensive PEDV antigen distribution in villous enterocytes, which was strongly correlated with the severity of tissue damage observed. These outcomes were comparable to those reported for highly virulent Korean G2b strains such as CKT-7, KNU141112-P5, and HS (50–52), as well as for Chinese G2c strains such as HM2017 and CH/JLDH/2016 (17, 18). The magnitude of clinical disease was comparable to that reported for previously described virulent Korean G2b and Chinese G2c strains. Given its genetic relatedness to Chinese highly virulent variants, KPED2021-1 may represent a lineage with similar pathogenic potential. From a disease control perspective, virulent PEDV strains currently identified in South Korea can be broadly categorized into two groups: the endemic G2b lineage that has been predominant in the country since its introduction around 2013, and the genetically distinct Chinese-like lineage identified in the present study. In China, PEDV variants belonging to similar genetic lineages have been reported to exhibit limited protection against existing commercial vaccines (53). In the present study, the outbreak occurred in a herd vaccinated with commercial G2b-based vaccines; however, antigenic relationships between KPED2021-1 and existing vaccine strains were not directly evaluated. It should be noted that vaccine performance against PEDV can also be influenced by vaccination strategy and route of administration. Oral vaccination is generally intended to stimulate mucosal immunity, particularly lactogenic secretory IgA, whereas intramuscular vaccination may primarily induce systemic antibody responses (54). Therefore, reduced protection observed in certain field situations may reflect differences in immune induction as well as viral genetic variation. Further studies, including cross-neutralization assays, will be necessary to determine the extent to which current vaccines provide protection against this genotype.

Several limitations of this study should be acknowledged. First, although multiple genetic variations were identified in the S gene, as well as in NSP1, ORF3, and the N gene, this study did not experimentally evaluate the functional consequences of these mutations on viral pathogenicity, immune evasion, or host–virus interactions. Second, while the phylogenetic analyses support a close genetic relationship between KPED2021-1 and Chinese PEDV variants, molecular data alone cannot precisely reveal the route or timing of viral introduction into South Korea. These limitations highlight the need for further studies incorporating expanded field surveillance, cross-neutralization assays to assess antigenic relationships, and reverse genetics-based functional analyses to clarify the biological significance of the observed genetic divergence.

## 5 Conclusion

This study is the first to isolate and comprehensively characterize a PEDV strain (KPED2021-1) in South Korea that is genetically distinct from previously circulating domestic G2b strains and closely related to Chinese PEDV variants. The isolate demonstrated virulent pathogenicity in experimentally infected suckling piglets, causing acute watery diarrhea, severe villous atrophy, and mortality. These findings confirm its enteropathogenic potential and highlight the ongoing genetic diversification of PEDV and its possible influence on disease severity in neonatal pigs. From an epidemiological perspective, the identification of a lineage closely related to strains circulating in China suggests regional genetic connectivity within East Asia, although the precise route and timing of introduction cannot be determined based solely on molecular data. These findings underscore the importance of sustained whole-genome surveillance, strengthened cross-regional data sharing, and periodic evaluation of vaccine–field strain compatibility to support evidence-based prevention and control strategies against emerging genetically divergent PEDV variants in South Korea.

## Data Availability

The datasets presented in this study can be found in online repositories. The names of the repository/repositories and accession number(s) can be found in the article/Supplementary material.
