# Supplementary material for: Isolation, genomic characterization, and pathogenicity of an emerging PEDV variant in Korea related to virulent Chinese strains
Source: Front Vet Sci. 2026 Apr 1;13:1785848. doi: 10.3389/fvets.2026.1785848 (PMC13079152; doi:10.3389/fvets.2026.1785848)
Supplement: Supplementary file 1 [file Data_Sheet_1.docx]

**
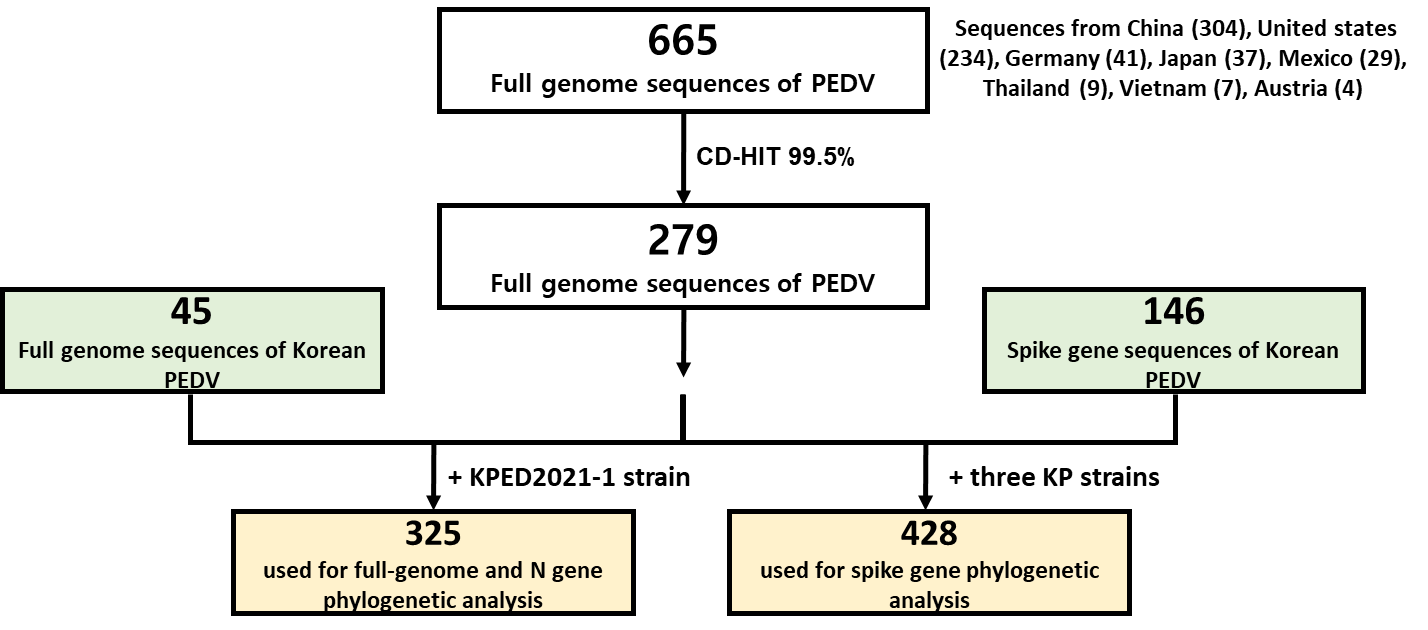
**

**FIGURE S1. Schematic Diagram of Sequence Collection and Selection for Phylogenetic Analysis of PEDV Strains** A total of 665 full-genome PEDV sequences were initially collected from various countries, including China (304), the United States (234), Germany (41), Japan (37), Mexico (29), Thailand (9), Vietnam (7), and Austria (4). Following sequence clustering analysis using CD-HIT with a 99.5% identity threshold, 279 representative sequences were selected. These sequences were subsequently integrated with 45 full-genome sequences and 146 spike gene sequences derived from Korean PEDV strains. The KPED2021-1 strain was added to create a dataset of 325 sequences for full-genome and N gene phylogenetic analysis. For spike gene phylogenetic analysis, the dataset was further expanded to 428 sequences by including three additional KP strains.


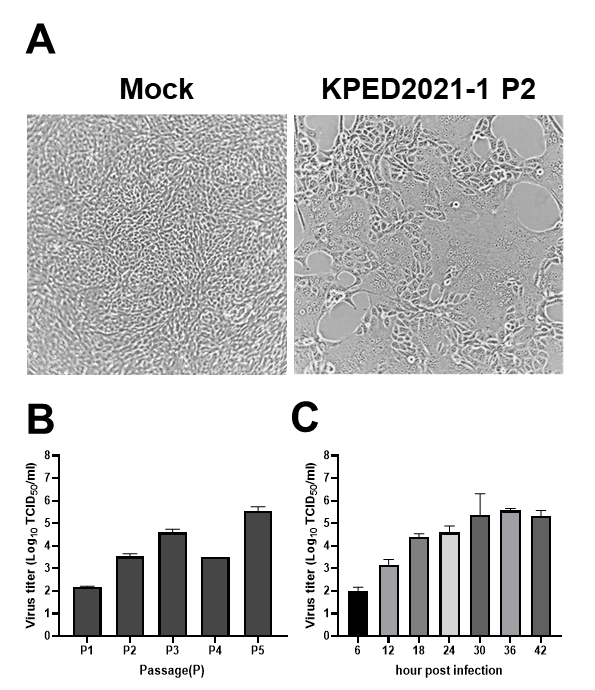


**FIGURE S2. Isolation of KPED2021-1 in Vero cells.** Cytopathic effects (CPE) were observed in Vero cells infected with KPED2021-1 at passage 2 (P2). Mock-infected cells served as a control. Images were captured at 24 hours post-infection using phase-contrast microscopy.

**TABLE S1.** Body weights and average daily weight gain (ADWG) of piglets in negative control and KPED2021-P5-infected groups.

| Group | Piglet no | Body weight (kg) | | | | ADWG  (g/day) |
| --- | --- | --- | --- | --- | --- | --- |
|  |  | 0 dpi | 5 dpi* | 6 dpi* | 7 dpi |  |
| Negative | NC-1 | 2.53 | - | - | 2.65 | 17.86 |
|  | NC-2 | 1.52 | - | - | 1.63 | 15.71 |
|  | NC-3 | 1.93 | - | - | 2.11 | 25.71 |
|  | NC-4 | 1.87 | - | - | 2.35 | 68.57 |
|  | NC-5 | 1.66 | - | - | 2.25 | 84.29 |
| KPED2021-P5-infected | P5-1 | 2.04 | 1.5 | - | - | -408.00 |
|  | P5-2 | 2.28 | 1.74 | - | - | -455.00 |
|  | P5-3 | 1.35 | 0.95 | - | - | -269.00 |
|  | P5-4 | 1.96 | - | - | 1.48 | -69.29 |
|  | P5-5 | 1.92 | - | 1.52 | - | -319.83 |

*Death occurred at 5 DPI (P5-1, P5-2, P5-3) and 6 DPI (P5-5). ADWG was calculated as weight gain divided by the number of survival days (g/day).
